# The influence of visual flow and perceptual load on locomotion speed

**DOI:** 10.3758/s13414-017-1417-3

**Published:** 2017-09-19

**Authors:** Casimir J.H. Ludwig, Nicholas Alexander, Kate L. Howard, Alicja A. Jedrzejewska, Isha Mundkur, David Redmill

**Affiliations:** 1 School of Experimental Psychology, University of Bristol, Bristol, UK; 2 Bristol Vision Institute, Bristol, UK; 3 School of Life and Health Sciences, Aston University, Aston, UK

**Keywords:** Locomotion, Visual flow, Self-motion, Dual-task, Perceptual load

## Abstract

Visual flow is used to perceive and regulate movement speed during locomotion. We assessed the extent to which variation in flow from the ground plane, arising from static visual textures, influences locomotion speed under conditions of concurrent perceptual load. In two experiments, participants walked over a 12-m projected walkway that consisted of stripes that were oriented orthogonal to the walking direction. In the critical conditions, the frequency of the stripes increased or decreased. We observed small, but consistent effects on walking speed, so that participants were walking slower when the frequency increased compared to when the frequency decreased. This basic effect suggests that participants interpreted the change in visual flow in these conditions as at least partly due to a change in their own movement speed, and counteracted such a change by speeding up or slowing down. Critically, these effects were magnified under conditions of low perceptual load and a locus of attention near the ground plane. Our findings suggest that the contribution of vision in the control of ongoing locomotion is relatively fluid and dependent on ongoing perceptual (and perhaps more generally cognitive) task demands.

**Electronic supplementary material:**

The online version of this article (10.3758/s13414-017-1417-3) contains supplementary material, which is available to authorized users.

## Introduction

Visual flow is an important perceptual cue that may be used in starting, stopping, and ongoing control of human and non-human locomotion (Gibson 1958; Lappe et al. 1999; Srinivasan and Zhang 2004). Visual flow provides information about the speed, direction, and distance of self-motion (Larish and Flach 1990; Srinivasan et al. 1996; Redlick et al. 2001; Warren et al. 2001; Baird et al. 2005; Durgin et al. 2005), and may be used to calibrate the relation between biomechanical activity and perceived or anticipated speed of self-motion (Rieser et al. 1995; Harris et al. 2000; Proffitt et al. 2003; Durgin et al. 2005).

Visual flow encompasses the ‘optical edge rate’ and the ‘global rate of optical flow’ (Larish and Flach 1990; Mohler et al. 2007; François et al. 2011). The edge rate refers to the rate at which features pass a reference point and is dependent on the density of the features in the environment. The global rate of optical flow refers to the angular motion of features on the retina of an observer and therefore varies with the distance and direction of those features relative to the observer. As different studies manipulate edge rate, global rate of optical flow, or both, we adopt the more general term ‘visual flow’ throughout this paper. Non-visual information of course also provides information about self-motion (e.g., L. R. Harris et al., 2000; see also the extensive literature on path integration, e.g., Mittelstaedt & Mittelstaedt 2001). For example, interoceptive signals about self-motion come from the vestibular system, proprioception, and efference copy.

In human locomotion on foot, previous studies have shown that visual cues to self-motion are involved in the online control of walking speed even in the presence of veridical non-visual cues (Pailhous et al. 1990; Prokop et al. 1997; Mohler et al. 2007; De Smet et al. 2009; François et al. 2011). The contribution of vision is typically assessed by creating cue conflicts between visual and non-visual sources of information about self-motion speed. By presenting moving patterns on the floor or walls (Pailhous et al. 1990; De Smet et al. 2009) or in a virtual environment (Prokop et al. 1997; Mohler et al. 2007; Chou et al. 2009; François et al. 2011), the physical walking speed may be dissociated from the speed indicated by the visual flow^1^. When the visual flow signals that the subject is walking faster or slower than (s)he really is, the subject will typically slow down or speed up respectively, as if (s)he is aiming to maintain a relatively constant rate of flow.

The usual relation between biomechanical activity and its expected multi-modal consequences is altered in cue-conflict scenarios, potentially resulting in perceptual-motor recalibration (Durgin et al. 2005) and/or a change in the weighting of the various cues to self-motion. For example, Campos et al. (2014) showed that the weighting of visual cues diminished when the visual gain of the visual flow was incongruent with the (veridical) non-visual cues. The authors argued that the precision or reliability of the manipulated cue in signaling self-motion decreases when considered over the course of a trial sequence (especially when different gains are randomly inter-mixed from trial to trial). As a result, a likely outcome is that the weight of the manipulated cue decreases. Moreover, when walking on a treadmill through a virtual environment, non-visual vestibular cues may be degraded compared to regular locomotion overground (Durgin et al. 2005). It is therefore an open question as to whether and to what extent visual flow influences overground locomotion speed in regular, static environments without cue conflicts between the available visual and non-visual cues.

In (simulated) driving, visual flow is clearly used to control the speed and direction of the vehicle (Denton 1980; Wilkie and Wann 2003; Kountouriotis et al. 2013). Of course when driving a vehicle, many of the usual non-visual cues to self-motion are absent and under such circumstances we would expect visual cues to be weighted highly (especially when the self-motion is simulated). Of particular relevance to the current study is that static textures may be used to induce an “illusion” of speed change. In a real-world experiment, (Denton 1980) demonstrated that in the approach to a roundabout decreasing the spacing of horizontal lines on the road led drivers to slow down. Drivers presumably interpreted the increase in visual flow as an increase in self-motion speed, a phenomenon referred to as ‘control of speed by illusion’. We were interested in whether such manipulations worked in regular locomotion on foot in the presence of veridical non-visual cues, partly with a view to potential application in the regulation of real-world locomotor behavior.

Such applied considerations also led us to probe the role of visual attention in the use of visual flow in locomotion. In regular locomotion, visual attention is typically directed to regions and objects of interest in the environment that have little to do with the regulation of ongoing self-motion speed (e.g., a traffic sign or shop window). As a result, most of the visual flow is visible in parafoveal and peripheral vision. In many of the studies investigating the link between visual flow and self-motion (perception) described above, participants attended to the visual motion, either because the task required it or because there was little else to see. It is unclear whether the use of visual flow in the regulation of self-motion requires the involvement of visual attention (or, indeed, the use of mental resources more generally). To address this question, we developed a dual-task paradigm in which a simple locomotion task was combined with a demanding perceptual discrimination task at a different location from the visual flow. The logic is that if the regulation of locomotion by visual flow requires attention, then drawing attention to some object in the environment should impact the influence of visual flow.

**Fig. 1:**
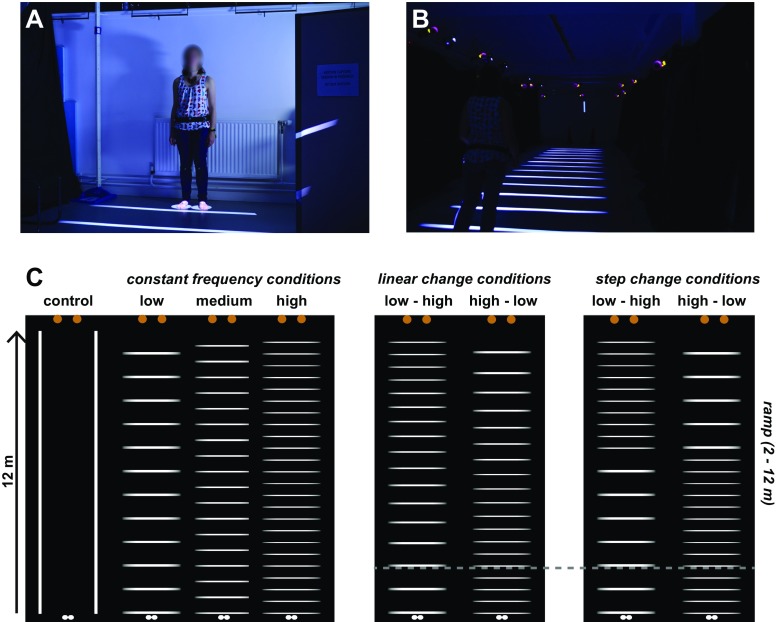
Illustration of the marker set up, the projected walkway and the different floor patterns. **a** Marker set up. Passive, infrared reflective markers were affixed to the waist, knees, and feet. Participants in the actual experiments wore shorts or tight-fitting clothing; this participant is one of the experimenters simply demonstrating the set up. The experimenter is standing on two projected floor spots that indicated the starting point. **b** Projected walkway with the discrimination target on the end wall. Two traffic cones signaled the end of the walkway, but these are not visible in this photograph. **c** Floor pattern profiles. The frequency was always constant in the first 2 m of the walkway. In the step-change condition, the frequency step occurs halfway over the range 2–12 m (i.e., at 7 m). The *gray dashed line* indicates the 2-m point, but was not visible to the participant. The *two orange dots* indicate the traffic cones at the end of the walkway

Figure 1 illustrates the paradigm. Participants walked across a 12-m projected walkway, with rectangular stripes oriented in the direction orthogonal to the walking direction. In the critical conditions (Fig. 1C, right four panels), we changed the spatial frequency of the floor pattern over the course of the walkway. The frequency either increased or decreased. If participants maintained a constant walking speed, visual flow from the floor texture would increase if the spatial frequency increased (and decrease if the spatial frequency decreased). If participants then used this change in the rate of flow to regulate their movement speed, we would expect them to slow down when the spatial frequency increased and to speed up when the spatial frequency decreased.

During their walk, participants had to perform a perceptual tilt discrimination of a stimulus presented on the wall near the end of the walkway (Fig. 1b). The stimulus was a bar with a small tilt offset from vertical. Importantly, the tilt offset was sampled sequentially from a Gaussian distribution and each sample was shown for 100 ms. As a result of the Gaussian noise, the best strategy for making this perceptual judgment is to integrate multiple samples in order to compute an average tilt offset over some epoch. Our assumption is that this task effectively forced participants to fixate and attend to the stimulus on the end wall for most, if not all of their walk. Once participants reached the end of the walkway, they verbally reported the direction of tilt offset (‘left’ or ‘right’ with regard to the top of the bar).

This perceptual task fulfilled several important functions. First, it mimicked real-world conditions in which visual attention is focused on objects that are unrelated to the ongoing locomotor task. Second, the implicit control of fixation ensured that the visual flow was mostly viewed in peripheral vision. Third, by manipulating the difficulty of the tilt discrimination task, we could vary the perceptual demands of this task and assess whether and to what extent the influence of visual flow depends on the amount of perceptual processing capacity available (Kahneman 1973; Pashler 1994; Wickens 2002; Tombu and Jolicoeur 2003; Lavie et al. 2004; Lavie et al. 2014). Our primary measure of interest was locomotor speed, which we measured in 2-m sections of the walkway.

## General methods

### Participants

Participants attended a single ∼1 h session, for which they were paid a fee. Across both experiments, 66 participants were tested in order to achieve a target sample size of 30 participants in each experiment. In Experiment 1, four participant were excluded due to problems in recording their data (stray reflections and missing marker data). In Experiment 2, two participants were excluded for not complying with the task instructions. Participants (35 women and 25 men in the final set) were recruited from the local population and had an average age of ∼25 years. Ethical clearance was obtained from the local Faculty of Science Human Research Ethics Committee. The experiments were conducted in accordance with the ethical guidelines of the British Psychological Society (which are in line with those of the APA). All participants provided written informed consent and were fully debriefed.

### Materials

Participants walked on a projected walkway and we measured their motion using passive, optical motion capture with a 12-camera Qualisys (AB, Götenborg, Sweden) System (Oqus 300 cameras), sampling the marker positions at 100 Hz. An array of six projectors (Optoma EW536, resolution 1280 × 800, frequency 60 Hz) was used to create the walkway by projecting onto the gray floor surface. The image for each projector was corrected to account for (i) the variation in luminance at different distances, and (ii) the overlapping projection region with the “neighboring” projector. A seventh projector (Optoma EW775) was used to project the discrimination target (a 50 × 6 cm tilted bar on the end wall).

Figure 1 demonstrates the marker set up on one of the experimenters (actual participants wore shorts or tight-fitting sports clothing to minimize extraneous marker motion). A belt attached to the waist with Velcro contained three markers; a further two markers were fixed just below the knees at the top of the tibia; another two markers were fixed to the first metatarsal of both feet. For the analysis of walking speed, we used the waist marker. Additional markers were included to allow for analysis of stride length and stride frequency (using the feet markers) and to aid automated identification of markers in the Qualisys Track Manager software (a more elaborate marker set introduces more constraints as to how different markers move relative to each other, which aids identification).

Each floor pattern spanned a region of 2 × 12 m (w ×l). A blank control pattern contained just two “tramlines” in the direction of locomotion. The tramlines (10 cm wide) were there to provide illumination and signal the width of the walkway. In Experiment 1, we also used three floor patterns with a constant spatial frequency: low (1 cycles/m), medium (1.5 cycles/m), and high (2 cycles/m). With increasing spatial frequency, the thickness of the bright stripes decreased to keep the overall luminance integrated across the walkway approximately constant. The remaining floor patterns changed in frequency either gradually (linear ramp) or suddenly (step). The change was either from a low to a high spatial frequency (increasing flow) or vice versa (decreasing flow). For these changing walkways, the first 2 m always had a constant frequency. The ramp or step was only implemented for the 2–12 m section of the walkway, with the step occurring halfway this section.

### Procedure

Participants were asked to attend the lab session wearing shorts or leggings. After they were given information about the study and had provided informed consent, they were asked to stand at the start of the walkway (indicated by two projected spots on the floor) and practice the perceptual discrimination task. This task involved a tilt offset judgement. A bar projected on the end wall changed orientation every 100 ms. The orientations were drawn from a Gaussian distribution with a mean offset from vertical of ±1^∘^ or ±2^∘^ (±1^∘^ in Experiment 1; ±1^∘^ or ±2^∘^ in Experiment 2), and a standard deviation of 6^∘^ (Ludwig et al. 2014). The nature of this task is such that any one “sample” of the bar does not provide sufficient information about its true tilt offset. Multiple samples have to be used for a more accurate perceptual decision. As such, we encouraged participants to maintain their attention and fixation on the end wall for at least most of their walk.

**Fig. 2:**
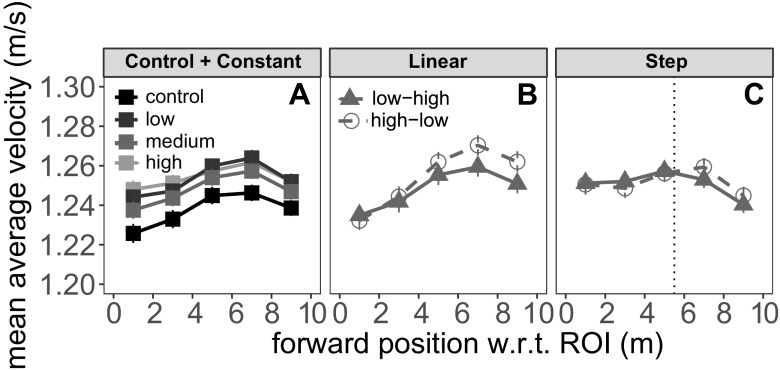
Walking speed as a function of position on the walkway. *Error bars* are within-subject standard errors of the mean(Morey 2008). The *vertical dotted line* in panel **c** shows the position of the step change on the walkway

**Fig. 3:**
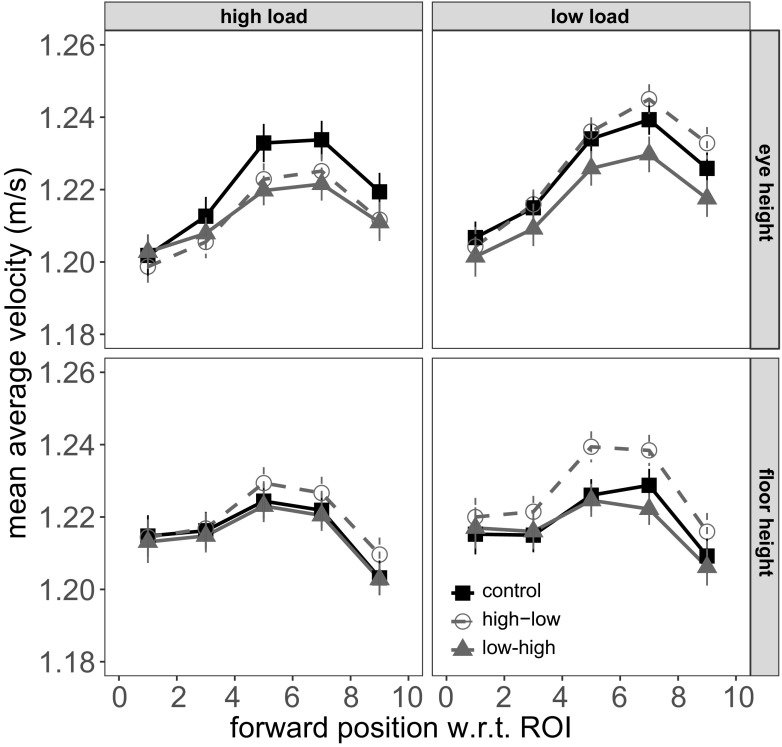
Walking speed as a function of position on the walkway. The different functions in each panel correspond to the different floor patterns (control and two directions of linearly changing spatial frequency). *Error bars* are within-subject standard errors of the mean

Once participants were comfortable with the perceptual discrimination task, we proceeded with the main locomotion experiment. Initially, the walkway was blank except for the two projected start markers. After a delay, the walkway appeared, along with a static vertical bar on the end wall. The onset of these patterns was the cue for participants to start walking. Half a second after the appearance of the walkway and the vertical bar, the bar started fluctuating around its mean orientation for 8 s. Participants walked at their own pace to the end of the walkway, marked by a pair of traffic cones. They verbally communicated their perceptual response to the experimenter (‘left’ or ‘right’ tilt for anticlockwise and clockwise, respectively) when they reached the traffic cones or upon the offset of the bar, whichever came later. Typically, participants reached the end of the walkway in less than 10 s (an average walking speed just over 1.2 m/s; see Figs. 2 and 3), around the same time as the end of the tilt animation.

### Data analyses

The *y*-axis of our measurement volume is aligned with the length of the walkway and only this dimension was used in the analyses reported below. Markers were identified using Qualisys Track Manager (Qualisys, AB) software, using their ‘Automated Identification of Markers’ model. The labeled data were exported to Matlab (Mathworks) and the (time, position) trajectories of the critical markers (waist, left, and right feet) were plotted for each trial to check the accuracy of the labeling.

Marker data were less reliably available near the start and the end of the walkway, that is, close to the edges of the capture volume. Therefore, we adopted the interval 1.5–11.5 m as our region of interest (ROI). To compute instantaneous walking speed, we computed the finite differential using one sample either side of the time point of interest (with the Matlab ‘gradient’ function). For the waist data, we then simply averaged the instantaneous velocity in five 2-m bins (centered at 1, 3, 5, 7, and 9 m after the start of the ROI). Any changes in walking speed will be mediated by changes in stride length and/or stride frequency (Laurent and Pailhous 1986). These secondary variables were computed from the feet marker data and are reported in the Supplementary Materials for Experiment 2.

The statistical assessment of the impact of our manipulations was performed by comparing linear mixed effects models with different (combinations of) predictor variables (Baayen et al. 2008; Baguley 2012). The sets of predictor variables differed between the two experiments and are described in detail in the Results section of each experiment. Mixed effects models are applied to the data from individual trials, which is more powerful than aggregating data across trials for each participant and then analyzing only participant means. This approach naturally deals with variable number of trials in different experimental conditions and the variable precision in the measurements extracted from those trials, which would be ignored when aggregating across trials.

In all of our analyses, a null model was defined, which consisted simply of a distribution of subject-specific intercepts (i.e., a different mean walking speed for each participant). Depending on the experimental manipulations, more complex models were then built that included one or more predictor variables (e.g., the nature of the floor pattern) and their interactions. If a predictor variable accounts for variance in the walking speed data, a model that includes that predictor will provide a better account of those data. However, models with more predictor variables necessarily account for more variance. The question is whether the improvement in goodness-of-fit is worth the additional model complexity introduced by including additional predictors. To this end, we used the Bayesian Information Criterion (BIC; Schwarz 1978, Raftery 1999, Wagenmakers 2007) to compare models with different variables. The BIC penalizes a deviance measure of goodness-of-fit (twice the negative log-likelihood) with a term that depends on the number of free parameters in the model and the amount of data. BICs tend to favor simpler models compared to other common statistical model comparison techniques (e.g., Akaike Information Criterion). In other words, BICs tend to be somewhat conservative in the assessment of the need for additional predictor variables. One reason we adopted this model selection approach to our statistical assessment is that it simply summarizes the evidence in favor of different models, which also allows us to make inferences in favor of, say, a null model.

## Experiment 1

Experiment 1 was designed to address the following questions. First, are there any differences in walking speed for patterns of different, but constant spatial frequencies? To address this question, we included low, medium, and high spatial frequency walkways in which the spatial frequency remained constant. For example, it is possible that participants might adjust their stride length to the spacing of the visual elements on the floor (i.e., smaller steps over high spatial frequency textures). Without adjustment of stride timing, such behavior would result in slower walking speeds over high spatial frequency walkways. If we then observed a slowing down of walking speed over walkways in which the spatial frequency changed from low to high, it would not be clear cut that such a change in walking speed is due to a change in *visual flow*. Second, does a change in spatial frequency (visual flow) influence walking speed and, if so, in what way does this effect depend on the spatial profile of the frequency change (step or linear ramp)? We expected walking speed to decrease if the spatial frequency changed from low to high, and to increase if the spatial frequency changed from high to low. We did not have strong predictions about the spatial profile of the frequency change.

All 30 participants experienced eight trials of the blank control and the three constant frequency conditions (Fig. 1C, left four panels). The spatial profile of the frequency change was manipulated between subjects to keep the overall number of trials limited to a single experimental session of around 1 h. The ‘Linear’ group consisted of 15 participants who were exposed to 12 trials of the two types of linear change profile. The ‘Step’ group consisted of 15 participants who were exposed to 12 trials of the step-change profile. Therefore, each participant performed 56 trials in total. These trials were randomly intermixed, independently for each participant. The mean tilt offset of the discrimination target was 1^∘^.

### Results and discussion

One participant scored below chance on the discrimination task (performing at 25% correct; we found out too late to ask the participant what (s)he was responding to), but the remaining scores averaged 78% correct (range, 54–91%). In other words, the perceptual discrimination task was difficult, but possible for the vast majority of participants.

Figure 2 illustrates the walking speed at different points on the walkway. Consider the control and constant frequency conditions (panel a) first. Participants first sped up and then slowed down as they approached the end of the walkway. Walking speed appears slowest overall in the control (tramlines) condition. As such, it appears that walking speed is not dependent on the spatial frequency of the floor pattern when it is kept constant across the entire trajectory. Such an effect might have been expected if participants, for example, adjusted their stride length to the line spacing (while keeping their stride frequency constant).

We assessed these four conditions statistically with the following linear models: (i) ‘null’ model with participant as a random factor (random intercept model); (ii) ‘position’ model, with position as a continuous predictor added to the null model; (iii) ‘pattern’ model, in which the nature of the floor pattern was added as a categorical predictor to the null model; (iv) ‘additive’ model, in which both predictors were included; (v) ‘full’ model, which included both predictors *and* their interaction.

Table 1 lists the BIC values for the different models, applied to different subsets of the data. Note that lower values indicate “better” models, but the overall scale of the values is largely determined by the amount of data. Therefore, only relative comparisons within a subset (i.e., within a column) are meaningful. BICs within a column were converted to weights that, under certain assumptions, may be viewed as approximations to the posterior probability of a model, given the data and the other models in the comparison set (Wasserman 2000; Wagenmakers 2007). These BIC weights sum to 1 across the set of models under consideration (in a column) and provide a more intuitive measure of the strength of support for a model.

**Table 1:** BIC values for different linear mixed effects models fit to the walking speed data from Experiment 1

**Model**	control and constant frequency	constant frequency	linear change	step change
Null (3)	-12470	-9525	-4432	**-4890**
Position (4)	-12484	**-9532**	**-4460**	-4883
Pattern (6/5/4/4)	-12495	-9516	-4427	-4882
Position + Condition (7/6/—/—)	**-12509**	-9522	—	—
Full (8/8/6/6)	-12485	-9507	-4451	-4870

The winning model, in terms of the lowest BIC, is indicated in *bold*. The ‘—’ represents that a model was not included in the comparison set. The number of free parameters for each model are given in *parentheses* (separately for the different columns, where necessary)

In the analysis of the control and constant frequency conditions, the BIC weight for the additive model was effectively 1 (> 0.99), so there is overwhelming evidence that both position and pattern matter. We repeated the analysis for the three constant frequency conditions only (i.e., control condition removed). Now the BIC weight for the ‘position’ model was ∼ 0.95, with the next best model being the ‘null’ model with a weight of ∼ 0.04. Overall then, these analyses suggest that participants walked more slowly in the blank control condition. In addition, their speed depended on the position on the walkway (speeding up near the beginning, slowing down near the end), but the nature of this speed change did not depend on the spatial frequency floor pattern. We have no evidence that different spatial frequencies, when held constant across the entire trajectory, influenced locomotor speed.

Figure 2b shows the velocity in the two linear change conditions. Walking speed had the same characteristic pattern of speeding up in the beginning and slowing down near the end. However, the functions seem to diverge for the two floor patterns: at the start of the walkway there was no difference in walking speed, but by the end participants in the ‘high-low’ condition were walking slightly faster. This profile is qualitatively consistent with a “relative speeding up” as the spatial frequency of the pattern decreases. Figure 2c shows the data from the two step-change conditions. While there is a slight hint of a divergence between the two conditions just after the frequency change, the shift is very small and the two functions largely lie on top of each other.

We adopted the same model comparison approach for the data from these conditions (separately for the ‘Linear’ and ‘Step’ groups). In this case, the additive model was not of prior interest, so we dropped this model from our set. That is, if visual flow modulated walking speed in these conditions, this should show up as an *interaction* between position and pattern, i.e., evidence for the full model. For the ‘Linear’ group, the best model was the ‘position’ model, with a BIC weight of effectively 1. The next best model was the ‘full’ model, but based on BICs this model was not competitive. For the ‘Step’ group, the best model was the ‘null’ model with a weight of ∼ 0.94. The next best model was the ‘position’ model with a weight of ∼ 0.04.

In summary, the data from the linear change conditions were qualitatively in agreement with the hypothesized changes in walking speed “by illusion” (Denton 1980). However, by the relatively conservative yardstick of BIC model selection, which heavily penalizes the addition of predictor variables, the influence of visual flow was not sufficiently large to warrant the additional complexity of including an interaction between position and floor pattern in our linear models. One hypothesis is that the effect of the floor pattern is small and simply needs a bigger sample to identify with our statistical model comparison approach. Another hypothesis is that the perceptual discrimination task was so demanding that there was relatively little spare capacity for using the visual flow to regulate walking speed. We addressed both these hypotheses in Experiment 2.

## Experiment 2

The primary aim of Experiment 2 was to assess locomotor behavior in the linear change condition in more detail. First, we tested a larger sample in this condition, so that we had more statistical power to detect the interaction between position on the walkway and the direction of the spatial frequency change. Second, we varied the difficulty of the perceptual tilt discrimination task, as a manipulation of perceptual load (Lavie et al. 2004; Lavie et al. 2014). As stated in the Introduction, it is possible that the processing of visual flow for the purpose of regulating self-motion, requires visual attention or mental resources more generally. If such resources are taken up with a demanding perceptual discrimination task, then reducing the load of that task may increase the influence of visual flow on locomotion. Note that this prediction is predicated on the assumptions that the (foveal) perceptual discrimination task and the (peripheral) processing of visual flow draw on the same “resource” at the same time, and that this resource has limited capacity (Kahneman 1973; Tombu and Jolicoeur 2003). Where these two tasks draw on different resources (e.g., foveal vs. “ambient” systems; cf. Wickens 2002), we would not expect variations in perceptual load to modulate the influence of visual flow on locomotion.

As a more exploratory manipulation, we varied the location of the perceptual discrimination target between groups of participants. The perceptual discrimination target could appear at eye height (as in Experiment 1) or near the bottom of the wall, i.e., near floor height. With a floor-height target, fixation should be focused on a distant point near the ground plane. As the walk progresses, the pattern of visual flow is then placed progressively closer to the fovea (Harris and Carré 2001). We assume that in this paradigm, with a demanding perceptual discrimination task, the locus of attention is tightly coupled to fixation, with both of them tied to the discrimination target (Shepherd et al. 1986; Sheliga et al. 1994; Deubel and Schneider 1996; Awh et al. 2006). The logic was that the influence of visual flow might be more pronounced when presented closer to the locus of fixation and attention (for a similar manipulation to assess the role of peripheral vision in stair descending, see Miyasike-daSilva & McIlroy 2016).

In Experiment 2, all participants experienced the two linear change conditions in addition to the (blank) control condition. The perceptual load manipulation was conducted within participants, as this was of key theoretical interest. In the high-load condition, the mean tilt offset was ±1^∘^; in the low-load condition, the mean tilt offset was ±2^∘^. The more exploratory manipulation of discrimination target height was conducted between subjects, in order to limit the total number of trials and the duration of the experiment to a single session of around 1 h. In the ‘Eye’ group, the height of the discrimination target was at 1.55 m, as in Experiment 1. In the ‘Floor’ group, the height of the target was 0.3 m. This manipulation lowers the discrimination target in the visual field by only a very small amount when standing at the start of the walkway, but progressively more towards the end of the walkway. For each participant there were ten trials for each combination of floor pattern (control, low-high, high-low) and perceptual load, for a total of 60 trials. These trials were randomly intermixed, independently for each participant. The experiment was followed up with a brief questionnaire to probe participants’ awareness of the aims of the experiment and its manipulations, in particular the variation in the floor patterns.

### Results and discussion

Across all conditions and participants, performance on the tilt discrimination task ranged between 60 and 100% correct. Average performance was 86% correct in the high-load condition and 90% in the low-load condition. Note that in comparison to Experiment 1, perceptual performance with a mean tilt offset of ±1^∘^ (high load condition here) was higher. In any event, performance was numerically better in the low load condition for 21/30 participants, and the overall effect size was 0.47 (Cohen’s *d*).

When asked to describe the different floor patterns, almost all participants referred to parallel horizontal (across) and vertical (tram) lines. When further probed in what way the patterns differed, one participant noted that the stripes got thinner towards the end of the walkway (which was true for the low-high condition). When told about the critical manipulation of spatial frequency, none of the participants correctly reported the purpose of this manipulation. Some participants reported that the floor patterns varied to see whether it would lead people to sway or veer and others reported that we were interested in the way the floor patterns affected the perceptual judgment of tilt. To conclude, apart from perhaps one participant, nobody explicitly noticed the spatial frequency manipulation in the critical conditions.

Figure 3 shows the walking speed as a function of position on the walkway. The different panels correspond to different combinations of perceptual load (columns) and discrimination target position (rows). We first assessed the overall influence of perceptual load and target position on walking speed, using the control data only and ignoring position on the walkway. These results are shown in Supplementary Fig. S1. We compared the following linear models: (i) ‘null’ (participant as a random factor); (ii) ‘load’ model, with perceptual load (low load, high load) as an additional predictor; (iii) ‘target’ model, with discrimination target height (eye, floor) as a predictor added to the null model; (iv) ‘load + target’ model, that included both predictor variables; and (v) ‘load ×target’ model, that also included the interaction between these two factors. The strongest model was the null model with a BIC weight of ∼ 0.92, followed by the load model (∼ 0.06) and the target model (∼ 0.02). Numerically, participants sped up slightly in the low-load condition, but this effect was small (Cohen’s *d* = 0.15). The overall effect size for target position was negligible (Cohen’s *d* = 0.08).

The key effect of interest in this experiment was, once again, the interaction between position on the walkway and the change in spatial frequency of the floor pattern. In addition, we were interested in the extent to which this interaction was modulated by perceptual load and discrimination target position. Therefore, we fit and compared the following linear models on the data from the two linear change conditions only: (i) ‘null’ (participant as a random factor); (ii) ‘position’ model; (iii) ‘pattern’ model; (iv) ‘position ×pattern’ model that included both main effects of position and floor pattern, as well as their interaction; (v) ‘position ×*pattern* ×load’ model that included the effect of load and its interaction with position and pattern; (vi) ‘position ×*pattern* ×target’ model that included the effect of discrimination target location and its interaction with position and pattern; (vii) ‘full’ model that included all four predictors and all their interactions. Table 2 lists the BIC values and corresponding weights for these different models.

**Table 2:** BIC values and their weights for different linear mixed effects models fit to the walking data from Experiment 2

**Model**	BIC	BIC weight
Null (3)	−16973	1.12 × 10^4^
Position (4)	−16985	5.73 × 10^2^
Pattern (4)	−16982	8.00 × 10^3^
Position × Pattern (6)	−**16990**	5.44 × 10^1^
Position × Pattern × Load (10)	−16988	2.15 × 10^1^
Position × Pattern × Target (10)	−16988	1.75 × 10^1^
Full (18)	−16956	1.90 × 10^8^

The winning model (lowest BIC, highest weight), is indicated in *bold*. The number of free parameters for each model are given in *parentheses*

The position ×pattern model had the lowest BIC and a weight ∼0.54. It was closely followed by the two three-way interaction models: the model that included perceptual load had a weight of ∼ 0.22 and the model that included target location had a weight of ∼ 0.18. All remaining models were far from competitive. Given the modulations observed in locomotor speed observed in this experiment, we assessed whether these effects were mediated by modification of the stride length or stride timing. Supplementary Figures 2 and 3 demonstrate that only stride length was similarly affected by the interaction between position, floor pattern, and perceptual load/target position. Therefore, it appears that speed changes were instantiated by changes in stride length, without modification of the stride timing (Prokop et al. 1997).

The winning position ×pattern model reflects the overall pattern shown in Fig. 3. Over the course of the walkway, the high-low and low-high conditions diverge, with relatively faster walking speeds in the high-low condition. This effect is consistent with the hypothesized influence of visual flow on locomotion speed. Moreover, the model selection results (Table 2) suggest that this pattern is influenced both by perceptual load and the position of the discrimination target.

**Fig. 4:**
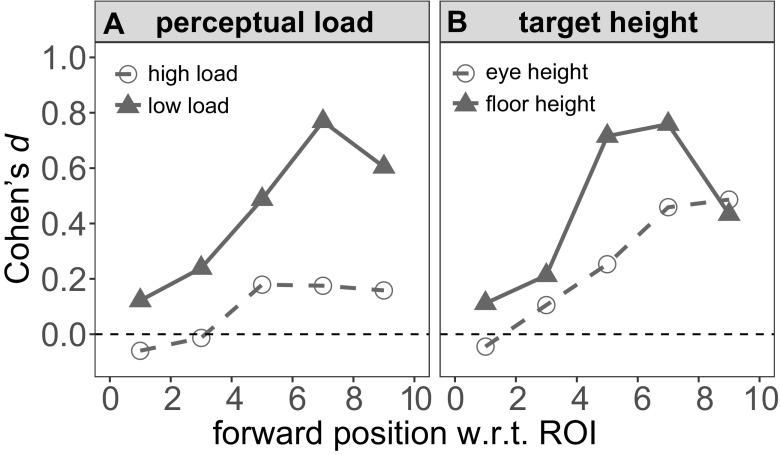
Floor pattern effect size as a function of position, separately for the two perceptual load conditions (**a**) and the position of the target (**b**). The effect size is the difference in mean velocity between the high-low and low-high floor patterns, normalized by the standard deviation of the difference scores

To assess these two three-way interaction models, we computed a floor pattern effect size for each positional bin. Figure 4a shows the effect size separately for the two load conditions. For this analysis, we pooled the data over the two target position groups and then for each positional bin and perceptual load condition, we computed the difference in mean velocity between the high-low and low-high floor patterns. The mean difference score was then normalized by the standard deviation of that difference score, to compute a Cohen’s *d* effect size. The results are clear: the effect of the floor pattern grows nonlinearly with position on the walkway, but grows to a much higher level in the low-load condition. Figure 4b shows the results from a similar analysis to assess the effect of target position on the modulation of walking speed by optic flow. Here, we pooled across the two load conditions and computed the floor pattern effect size separately for the ‘Eye’ and ‘Floor’ groups. Again, the effect of floor pattern emerges over the course of the walkway. However, the effect is much more pronounced when the discrimination target is near the ground.

## General discussion

We have demonstrated that static floor patterns with changing spatial frequencies can modulate walking speed in regular, overground locomotion when visual and non-visual cues to self-motion are all congruent. Moreover, this effect depended on concurrent perceptual load, with the floor patterns having a more pronounced effect when perceptual load was relatively low. There was also evidence that attending to a distant point near the ground plane increased the influence of visual flow arising from the ground plane.

Previous demonstrations of the effect of visual flow on locomotion speed generally decoupled visual information from non-visual sources of information about self-motion speed (Pailhous et al. 1990; Prokop et al. 1997; Mohler et al. 2007; Chou et al. 2009; De Smet et al. 2009; François et al. 2011; Campos et al. 2014). Such situations of cue conflict may (i) induce perceptual-motor recalibration and (ii) alter the weight associated with visual flow in the perception of self-motion, and thereby in the regulation of self-motion speed (Campos et al. 2014). The aim of this study was to assess the influence of visual flow on walking speed in regular, overground locomotion in which the various cues to self-motion were congruent. Part of our motivation for addressing this question was driven by the potential application of visual flow manipulations to regulate pedestrian behavior (e.g., influence their speed without obvious manipulations of the physical properties of the ground surface or through obstacles). In these situations, it is rarely practical to present adaptively moving patterns of flow that induce cue conflicts in the perception of self-motion. Therefore, we explored the notion of control over speed “by illusion” (Denton 1980).

In this context it should be noted that the effect of the floor patterns was numerically small (albeit robust, with peak effect sizes of *d* = 0.8; see Fig. 4), much smaller than the ∼ 10% effects reported in previous studies that involved strong cue conflicts (e.g. Prokop et al., 1997; Mohler et al., 2007; François et al., 2011). In our study, an “illusory” cue conflict would arise only if the visual system assumed that the spatial frequency of the floor pattern remained constant. Under that assumption, a change in visual flow is attributed to the participant’s own movement, rather than a change in the environment. Subjective reports indicated that the change in the environment was not noticed. However, that does not necessarily mean that the visual system attributed the change in spatial frequency/visual flow to a change in self-motion. Indeed, a number of cues were available that veridically signaled self-motion speed. In addition to the obvious non-visual cues, some of the visual information still specified movement speed accurately. For instance, the global rate of optic flow depends on the angular retinal motion of textural features in the environment and this rate is independent of the texture density (e.g., Larish & Flach, 1990; Fajen, 2005; François et al., 2011). Moreover, while we made an attempt to minimize extraneous visual features in the environment, some stable features remained visible (as shown in Fig. 1). As such, only the edge rate from a restricted part of the room (i.e., the ground plane) *could* be interpreted as a change in self-motion speed. In light of these considerations, obtaining *any* effect of visual flow on regular, overground locomotion on a short walkway, is remarkable.

The effect of visual flow was modulated by its position in the visual field and by concurrent perceptual load. Neither manipulation in itself had a major effect on locomotion speed, as assessed in the control condition of Experiment 2 (Fig. S1). If, for example, the position of the discrimination target induced a more or less natural (head) posture in one of the conditions, we might have expected locomotor speed to differ between the two target positions. Similarly, if participants responded to the perceptual load manipulation by minimizing their head motion in the more difficult, high-load condition, we might have expected an overall load effect on locomotor speed. There was little evidence for such effects. We now turn to the interaction of these factors with visual flow.

The variation in discrimination target height changed the retinal location of the visual flow: with a low discrimination target, the flow would have been positioned progressively closer to the fovea over the course of the walkway. We cannot differentiate whether it is the retinal eccentricity of the flow that matters or its position relative to the locus of attention. In both cases, greater proximity (low discrimination target) might enhance the influence of the flow. We did not record gaze, but our assumption is that fixation and attention are strongly linked (Shepherd et al. 1986; Sheliga et al. 1994; Deubel and Schneider 1996; Awh et al. 2006) and that both were maintained on the discrimination target for the vast majority of the walk. This assumption is plausible given that (i) the perceptual discrimination target was challenging and accuracy was clearly below ceiling, and (ii) the walk was over even, flat terrain with no obstructions, so there was not much need to acquire foveal information from the floor.

Peripheral vision is clearly involved in the control of locomotion. Making peripheral visual information more available facilitates the negotiation of obstacles (Graci et al. 2010) and stairs (Miyasike-daSilva and McIlroy 2016). Therefore, it is possible that with a discrimination target near floor level the visual flow was simply more clearly visible due to its greater proximity to the fovea (and/or the locus of attention). Moreover, the greater eccentricity of the visual flow with the discrimination target at eye height may have been compounded by a reduction in the useful field of view (around the discrimination target) induced by the locomotor task itself (Reed-Jones et al. 2014). Finally, it is also possible that with a low discrimination target, participants were able to switch covert and/or overt attention more easily between the discrimination target and the flow from the floor. More work is needed to assess the robustness of the effect of discrimination target height and identify the underlying mechanisms, particularly given the largely exploratory nature of this manipulation.

The effect of perceptual load is consistent with the view that processing the visual flow for the purpose of regulating self-motion speed requires mental capacity, that this capacity is limited, and that the perceptual tilt discrimination task also draws on this capacity (Kahneman 1973). The limited capacity mental resource in question is often equated with attention (e.g., Lavie et al., 2014). If the discrimination task is relatively easy, participants may be able to direct their attention to other parts of the environment. For example, they could rapidly switch attention between the discrimination target and the floor more frequently (Woodman and Luck 1999). Alternatively, they could switch their attention only once from the discrimination target to the floor, upon reaching some criterion amount of evidence about the direction of the tilt offset. In the low-load condition, this evidence criterion would be reached earlier (Gold and Shadlen 2001; Smith and Ratcliff 2004). Finally, it is possible that information from the floor and the discrimination target was processed in parallel, either through divided attention or through a flexibly configured attentional window that encompassed both the target and the floor (Eriksen and Yeh 1985; Jans et al. 2010). Either way, in the low-load condition, the division of attention or the flexible configuration of the attentional window could have been easier to achieve (Eriksen and St. James 1986; Lavie et al. 2004; Kyllingsbæk et al. 2011; Lavie et al. 2014). Regardless of the precise mechanisms involved, if more attention is allocated to the visual flow under conditions of low perceptual load, we might expect only modest improvements in perceptual discrimination performance in the low-load condition, as we observed.

It is important to note that processing the visual flow was not defined as an explicit task in our study. In a walking study, the regulation of speed and stability is of course an implicit task and it is likely that participants use various, if not all, available cues for this purpose. In our view, the most parsimonious explanation of the interaction between visual flow and perceptual load is that under conditions of low perceptual load, peripheral visual information from the ground plane was simply allowed to progress to higher levels of visual processing, much in the same way as the visual system “cannot help” but process (irrelevant) peripheral distractors in flanker interference tasks when central perceptual load is low (Eriksen and Eriksen 1974; Lavie et al. 2004; Kyllingsbæk et al. 2011). This view is also reminiscent of “tunnel vision” (Mackworth 1965; Ikeda and Takeuchi 1975), in which *relevant* peripheral information is processed less efficiently in the presence of a demanding foveal task (see Reed-Jones et al., 2014, for a similar phenomenon in a locomotion context). When the visual flow is allowed to progress within the visual system, it will be used as one of several cues that contribute to the regulation of self-motion.

Many studies have adopted a dual-task approach to assess the cognitive demands of maintaining postural stability and locomotion (for reviews, see Woollacott & Shumway-Cook, 2002; Yogev-Seligmann, Hausdorff, & Giladi, 2008; Al-Yahya et al., 2011). In most of the studies in this area, the secondary task is typically not visual (for exceptions, see for example, Sparrow, Bradshaw, Lamoureux, & Tirosh, 2002; Faulkner et al., 2006; Patel, Lamar, & Bhatt, 2014; Miyasike-daSilva & McIlroy, 2016). Frequently used tasks probe working memory (e.g., Ebersbach, Dimitrijevic, & Poewe, 1995; Springer et al., 2006; Hollman, Kovash, Kubik, & Linbo, 2007), auditory reaction time (e.g. Lajoie, Teasdale, Bard, & Fleury, 1993; Faulkner et al., 2006), verbal fluency (e.g., Harley, Wilkie, & Wann, 2009; Yogev-Seligmann et al., 2010; Patel et al., 2014), among others. Moreover, these studies are generally concerned with the contrast between the presence or absence of a dual task, rather than different levels of secondary task load. The conclusion that emerges from this previous work is that locomotion involves higher-level cognitive control, particularly when the locomotor task is challenging due to the nature of the terrain (e.g., the presence of obstacles) or due to the condition of the participant (e.g., ageing or neurological disease). The attentional demands are sometimes linked to the requirement to maintain stability (e.g., auditory RTs are increased during the single leg support phase in locomotion; see Lajoie et al., 1993). However, on the whole, our understanding of what aspects of locomotion are attentionally demanding is still very limited.

We did not include a “single-task” condition in which participants only had to complete the walking task. As such, we do not know what the basic effect of introducing the concurrent perceptual tilt discrimination task was on locomotor behavior. Given the unchallenging terrain, the use of a young and healthy participant sample, and the use of a basic discrimination or decision-making task, we would not expect the presence of the concurrent task itself to have a major effect on locomotion (Woollacott and Shumway-Cook 2002; Al-Yahya et al. 2011). Manipulating the load of the concurrent task did not really affect basic locomotor behavior (as evidenced by the control data of Experiment 2; Figure S1). Instead, the perceptual load effect was specific to the utilization of visual information for the control of self-motion, suggesting that this is one component of locomotion that requires attentional capacity. In this instance, it is possible that both “tasks” (processing visual flow and tilt discrimination) loaded perceptual mechanisms involved in the spatiotemporal integration of visual information (Kavcic and Duffy 2003). It remains to be seen whether the interaction of load with flow processing is specific to perceptual tasks that require spatiotemporal visual integration, or whether it generalizes to other forms of cognitive load (e.g., verbal fluency). In any event, our findings suggest that the weight of visual information to the perception and regulation of self-motion is relatively fluid and dependent on concurrent information processing demands.

## Electronic supplementary material

Below is the link to the electronic supplementary material.

(PDF 447 KB)

## Footnotes

1 Technically, there is no cue conflict in the “real-world” experiments in which visual motion is added to the scene by projecting on walls and ceilings, *only if* participants were perfectly able to decompose the retinal motion pattern into environmental and ego-motion components. However, judgements of self-motion under such conditions indicate that participants are not able to achieve veridical decomposition of visual motion signals.

## References

[CR1] Al-Yahya E, Dawes H, Smith L, Dennis A, Howells K, Cockburn J (2011). Cognitive motor interference while walking: a systematic review and meta-analysis. Neuroscience Biobehavioral Reviews.

[CR2] Awh E, Armstrong KM, Moore T (2006). Visual and oculomotor selection: links, causes and implications for spatial attention. Trends in Cognitive Sciences.

[CR3] Baayen RH, Davidson DJ, Bates DM (2008). Mixed-effects modeling with crossed random effects for subjects and items. Journal of Memory and Language.

[CR4] Baguley T (2012). Serious stats: A guide to advanced statistics for the behavioral sciences.

[CR5] Baird E, Srinivasan MV, Zhang S, Cowling A (2005). Visual control of flight speed in honeybees. Journal of Experimental Biology.

[CR6] Campos JL, Butler JS, Bülthoff HH (2014). Contributions of visual and proprioceptive information to travelled distance estimation during changing sensory congruencies. Experimental Brain Research.

[CR7] Chou Y-H, Wagenaar RC, Saltzman E, Giphart JE, Young D, Davidsdottir R, Cronin-Golomb A (2009). Effects of optic flow speed and lateral flow asymmetry on locomotion in younger and older adults: a virtual reality study. The Journals of Gerontology Series B: Psychological Sciences and Social Sciences.

[CR8] Denton GG (1980). The influence of visual pattern on perceived speed. Perception.

[CR9] De Smet K, Malcolm P, Lenoir M, Segers V, De Clercq D (2009). Effects of optic flow on spontaneous overground walk-to-run transition. Experimental Brain Research.

[CR10] Deubel H, Schneider W (1996). Saccade target selection and object recognition: Evidence for a common attentional mechanism. Vision Research.

[CR11] Durgin FH, Gigone K, Scott R (2005). Perception of visual speed while moving. Journal of Experimental Psychology: Human Perception and Performance.

[CR12] Durgin FH, Pelah A, Fox LF, Lewis J, Kane R, Walley KA (2005). Self-motion perception during locomotor recalibration: More than meets the eye. Journal of Experimental Psychology: Human Perception and Performance.

[CR13] Ebersbach G, Dimitrijevic MR, Poewe W (1995). Influence of concurrent tasks on gait: a dual-task approach. Perceptual and Motor Skills.

[CR14] Eriksen BA, Eriksen CW (1974). Effects of noise letters upon the identification of a target letter in a nonsearch task. Attention, Perception & Psychophysics.

[CR15] Eriksen C, Yeh Y (1985). Allocation of attention in the visual field. Journal of Experimental Psychology: Human Perception and Performance.

[CR16] Eriksen CW, St. James JD (1986). Visual attention within and around the field of focal attention: A zoom lens model. Perception Psychophysics.

[CR17] Fajen BR (2005). Calibration, information, and control strategies for braking to avoid a collision. Journal of Experimental Psychology: Human Perception and Performance.

[CR18] Faulkner KA, Redfern MS, Rosano C, Landsittel DP, Studenski SA, Cauley JA (2006). Reciprocal influence of concurrent walking and cognitive testing on performance in older adults. Gait Posture.

[CR19] François M, Morice AH, Bootsma RJ, Montagne G (2011). Visual control of walking velocity. Neuroscience Research.

[CR20] Gibson J (1958). Visually controlled locomotion and visual orientation in animals. British Journal of Psychology.

[CR21] Gold JI, Shadlen MN (2001). Neural computations that underlie decisions about sensory stimuli. Trends in Cognitive Sciences.

[CR22] Graci V, Elliott DB, Buckley JG (2010). Utility of peripheral visual cues in planning and controlling adaptive gait. Optometry Vision Science.

[CR23] Harley C, Wilkie RM, Wann JP (2009). Stepping over obstacles: attention demands and aging. Gait Posture.

[CR24] Harris LR, Jenkin M, Zikovitz DC (2000). Visual and non-visual cues in the perception of linear self-motion. Experimental Brain Research.

[CR25] Harris MG, Carré G (2001). Is optic flow used to guide walking while wearing a displacing prism?. Perception.

[CR26] Hollman JH, Kovash FM, Kubik JJ, Linbo RA (2007). Age-related differences in spatiotemporal markers of gait stability during dual task walking. Gait Posture.

[CR27] Ikeda M, Takeuchi T (1975). Influence of foveal load on the functional visual field. Perception Psychophysics.

[CR28] Jans B, Peters J, De Weerd P (2010). Visual spatial attention to multiple locations at once: The jury is still out. Psychological Review.

[CR29] Kahneman D (1973). Attention and effort.

[CR30] Kavcic V, Duffy CJ (2003). Attentional dynamics and visual perception: Mechanisms of spatial disorientation in Alzheimer’s disease, Vol. 126.

[CR31] Kountouriotis GK, Shire KA, Mole CD, Gardner PH, Merat N, Wilkie RM (2013). Optic flow asymmetries bias high-speed steering along roads. Journal of Vision.

[CR32] Kyllingsbæk S, Sy JL, Giesbrecht B (2011). Understanding the allocation of attention when faced with varying perceptual load in partial report: A computational approach. Neuropsychologia.

[CR33] Lajoie Y, Teasdale N, Bard C, Fleury M (1993). Attentional demands for static and dynamic equilibrium. Experimental Brain Research.

[CR34] Lappe M, Bremmer F, Van den Berg AV (1999). Perception of self-motion from visual flow. Trends in Cognitive Sciences.

[CR35] Larish JF, Flach JM (1990). Sources of optical information useful for perception of speed of rectilinear self-motion. Journal of Experimental Psychology: Human Perception and Performance.

[CR36] Laurent M, Pailhous J (1986). A note on modulation of gait in man: effects of constraining stride length and frequency. Human Movement Science.

[CR37] Lavie N, Beck DM, Konstantinou N (2014). Blinded by the load: attention, awareness and the role of perceptual load. Phil. Trans. R. Soc. B.

[CR38] Lavie N, Hirst A, De Fockert JW, Viding E (2004). Load theory of selective attention and cognitive control. Journal of Experimental Psychology: General.

[CR39] Ludwig CJ, Davies JR, Eckstein MP (2014). Foveal analysis and peripheral selection during active visual sampling. Proceedings of the National Academy of Sciences.

[CR40] Mackworth NH (1965). Visual noise causes tunnel vision. Psychonomic Science.

[CR41] Mittelstaedt M-L, Mittelstaedt H (2001). Idiothetic navigation in humans: Estimation of path length. Experimental Brain Research.

[CR42] Miyasike-daSilva V, McIlroy WE (2016). Gaze shifts during dual-tasking stair descent. Experimental Brain Research.

[CR43] Mohler B, Thompson W, Creem-Regehr S, Pick H, Warren W (2007). Visual flow influences gait transition speed and preferred walking speed. Experimental Brain Research.

[CR44] Morey RD (2008). Confidence intervals from normalized data: A correction to Cousineau (2005). Tutorial in Quantitative Methods for Psychology.

[CR45] Pailhous J, Ferrandez A-M, Flückiger M, Baumberger B (1990). Unintentional modulations of human gait by optical flow. Behavioural Brain Research.

[CR46] Pashler H (1994). Dual-task interference in simple tasks: Data and theory. Psychological Bulletin.

[CR47] Patel P, Lamar M, Bhatt T (2014). Effect of type of cognitive task and walking speed on cognitive-motor interference during dual-task walking. Neuroscience.

[CR48] Proffitt DR, Stefanucci J, Banton T, Epstein W (2003). The role of effort in perceiving distance. Psychological Science.

[CR49] Prokop T, Schubert M, Berger W (1997). Visual influence on human locomotion modulation to changes in optic flow. Experimental Brain Research.

[CR50] Raftery AE (1999). Bayes factors and BIC. Sociological Methods Research.

[CR51] Redlick FP, Jenkin M, Harris LR (2001). Humans can use optic flow to estimate distance of travel. Vision Research.

[CR52] Reed-Jones JG, Reed-Jones RJ, Hollands MA (2014). Is the size of the useful field of view affected by postural demands associated with standing and stepping?. Neuroscience Letters.

[CR53] Rieser JJ, Pick HL, Ashmead DH, Garing AE (1995). Calibration of human locomotion and models of perceptual-motor organization. Journal of Experimental Psychology: Human Perception and Performance.

[CR54] Schwarz G (1978). Estimating the dimensions of a model. Annals of Statistics.

[CR55] Sheliga BM, Riggio L, Rizzolatti G (1994). Orienting of attention and eye movements. Experimental Brain Research.

[CR56] Shepherd M, Findlay JM, Hockey RJ (1986). The relationship between eye movements and spatial attention. The Quarterly Journal of Experimental Psychology.

[CR57] Smith PL, Ratcliff R (2004). Psychology and neurobiology of simple decisions. Trends in Neurosciences.

[CR58] Sparrow W, Bradshaw EJ, Lamoureux E, Tirosh O (2002). Ageing effects on the attention demands of walking. Human Movement Science.

[CR59] Springer S, Giladi N, Peretz C, Yogev G, Simon ES, Hausdorff JM (2006). Dual-tasking effects on gait variability: The role of aging, falls, and executive function. Movement Disorders.

[CR60] Srinivasan M, Zhang S (2004). Visual motor computations in insects. Annual Review of Neuroscience.

[CR61] Srinivasan M, Zhang S, Lehrer M, Collett T (1996). Honeybee navigation en route to the goal: Visual flight control and odometry. The Journal of Experimental Biology.

[CR62] Tombu M, Jolicoeur P (2003). A central capacity sharing model of dual-task performance. Journal of Experimental Psychology: Human Perception and Performance.

[CR63] Wagenmakers E-J (2007). A practical solution to the pervasive problems of *p* values. Psychonomic Bulletin Review.

[CR64] Warren WH, Kay BA, Zosh WD, Duchon AP, Sahuc S (2001). Optic flow is used to control human walking. Nature Neuroscience.

[CR65] Wasserman L (2000). Bayesian model selection and model averaging. Journal of Mathematical Psychology.

[CR66] Wickens CD (2002). Multiple resources and performance prediction. Theoretical Issues in Ergonomics Science.

[CR67] Wilkie R, Wann J (2003). Controlling steering and judging heading: Retinal flow, visual direction, and extraretinal information. Journal of Experimental Psychology: Human Perception and Performance.

[CR68] Woodman GF, Luck SJ (1999). Electrophysiological measurement of rapid shifts of attention during visual search. Nature.

[CR69] Woollacott M, Shumway-Cook A (2002). Attention and the control of posture and gait: a review of an emerging area of research. Gait Posture.

[CR70] Yogev-Seligmann G, Hausdorff JM, Giladi N (2008). The role of executive function and attention in gait. Movement Disorders.

[CR71] Yogev-Seligmann G, Rotem-Galili Y, Mirelman A, Dickstein R, Giladi N, Hausdorff JM (2010). How does explicit prioritization alter walking during dual-task performance? Effects of age and sex on gait speed and variability. Physical Therapy.

