# Supplementary material for: The influence of visual flow and perceptual load on locomotion speed
Source: Atten Percept Psychophys. 2017 Sep 19;80(1):69–81. doi: 10.3758/s13414-017-1417-3 (PMC5735212; doi:10.3758/s13414-017-1417-3)
Supplement: Supplementary file 1 — (PDF 447 KB) [file 13414_2017_1417_MOESM1_ESM.pdf]

Supplementary Materials: The influence of visual flow and perceptual load on locomotion speed

Casimir J.H. Ludwig, Nicholas Alexander, Kate L. Howard, Alicja A. Jedrzejewska, Isha Mundkur, David Redmill

Influence of perceptual load and target height on locomotion speed

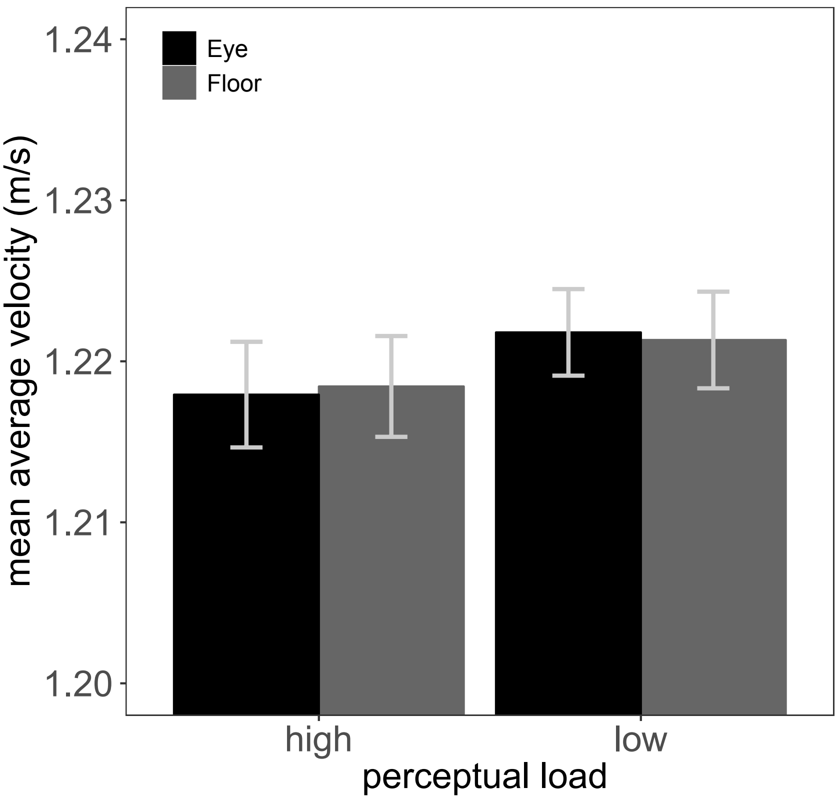

Figure S1: Average mean velocity as a function of perceptual load and discrimination target height in the blank control condition of Experiment 2. Velocity is computed across the entire Region of Interest. Error bars are within-subject standard errors of the mean. This figure (and the associated analyses reported in the main text) demonstrate that the manipulation of load and target height per se had minimal effects on locomotion.

Analysis of stride length and stride duration for Experiment 2

Experiment 2 reported a reliable effect of visual flow on locomotion speed, and this influence was modulated by perceptual load and discrimination target height. We assessed to what extent these speed changes were mediated by modulation of the stride length and the stride duration.

To analyses these variables, we selected the right foot by default, or the left foot if more data were available from that foot. Gaps of less than 350 ms were filled using spline interpolation over the whole trajectory, but the interpolated sample values were only used for the duration of the gap(s). We determined the maximum gap size we could fill by taking intact position-over-time trajectories and inserting artificial gaps of different sizes. This approach allowed comparison between the filled position values and the "true" position values. A cut-off value of 350 ms was chosen based on inspection of the figure relating error as a function of gap duration. Every trial in which one or more gaps were filled, was inspected and rejected if the filled trajectory appeared inconsistent with the surrounding samples. The gap-filled data were then low-pass filtered with a Butterworth filter of order 2 and a cut-off frequency of 10 Hz. To compute the instantaneous velocity of the foot, the filtered data were differentiated numerically in the same way as we did for the waist marker in the analysis of walking speed.

Strides were identified as follows. First, we identified the "stance phases" for the foot on a given trial as periods during which the foot was stable on the ground, as indicated by its forward velocity. The velocity criterion was set adaptively for each participant and each trial, based on the standard deviation of the velocities observed within that trial (Engbert & Kliegl, 2003)[1]. The velocity threshold was set at 0.25 times the standard deviation of velocities.

We identified points where the velocity dropped below threshold as the onset of a stance (offset of a stride). Points where the velocity increased above threshold correspond to the offset of the stance (onset of a stride). We applied a minimum duration criterion of 250 ms for the stance. If a stance met this duration criterion, we computed the stance position by taking the median of the position samples that made up the stance. The stride length is then the difference between successive stance positions, subject to a minimum of 0.2 m. The stride duration is the period of time between the offset of one stance and the onset of the next stance. The offset and onset points are defined by the points at which the velocity threshold is crossed. Identified strides were inspected visually by the analyst for each trial.

Figures S2 and S3 plot the stride length and stride duration in a similar format to the overall speed data shown in Figure 3 in the main text. It would appear that there is some periodic modulation of stride duration over the course of the walkway. We do not have an explanation for such modulation. Nevertheless, the important point is that for stride duration, the functions for the linear change condition do not systematically diverge in a manner that mirrors the changes in locomotion speed shown in Figure 3 of the main text.

[1]For the purpose of the present study, the consequences of such an adaptive criterion are minimal because the walking speed varies only over a very small range. This adaptive procedure would result in radically different thresholds for trials in which the participant ran vs shuffled, for example.

However, stride length shows a similar divergence between these two critical conditions to locomotion speed. That is, over the course of the walkway, there is a relative increase in stride length when the spatial frequency of the floor pattern decreases, compared to the condition in which the spatial frequency increases. In addition, these stride length modulations appear exaggerated under conditions of low perceptual load and with a discrimination target near the floor.

We analysed these data using exactly the same set of mixed effects models that were used to assess locomotion speed in Experiment 2. The BIC values for the models fit to the stride length and stride duration separately, are given in Table S1. For stride length, the Position×Pattern model was the winner (weight ~ 0.72), followed by the two three way interaction models. For stride timing, the simple Position-only model had the lowest BIC (weight ~ 0.98), suggesting that stride duration varied over the course of the walkway, but this effect was not influenced by any of the other variables. The similarity in the set of competitive models for velocity and stride length strongly suggests that any effects of visual flow, perceptual load and target location were predominantly mediated by adjustment of the stride length.

Table S1: BIC values for different linear mixed-effects models fit to the stride length and stride duration data from Experiment 2 (linear change conditions only). BIC weights are given in parentheses after the BIC values. The winning model (lowest BIC, highest weight), is indicated in bold. The number of free parameters for each model are given in parentheses after its descriptor.

**Model**	stride length	stride duration
Null (3)	-29449 (1.73×10^{-11})	-34011 (9.64×10^{-9})
Position (4)	-29481 (1.46×10^{-4})	**-34048** (9.78×10^{-1})
Pattern (4)	-29469 (4.43×10^{-7})	-34007 (1.52×10^{-9})
Position×Pattern (6)	**-29498** (7.19×10^{-1})	-34037 (4.28×10^{-3})
Position×Pattern×Load (10)	-29493 (4.95×10^{-2})	-34040 (1.77×10^{-2})
Position×Pattern×Target (10)	-29496 (2.31×10^{-1})	-34026 (2.30×10^{-5})
Full (18)	-29472 (1.44×10^{-6})	-34000 (5.75×10^{-11})

References

Engbert, R., & Kliegl, R. (2003). Microsaccades uncover the orientation of covert attention. *Vision Research*, *43*, 1035–1045.

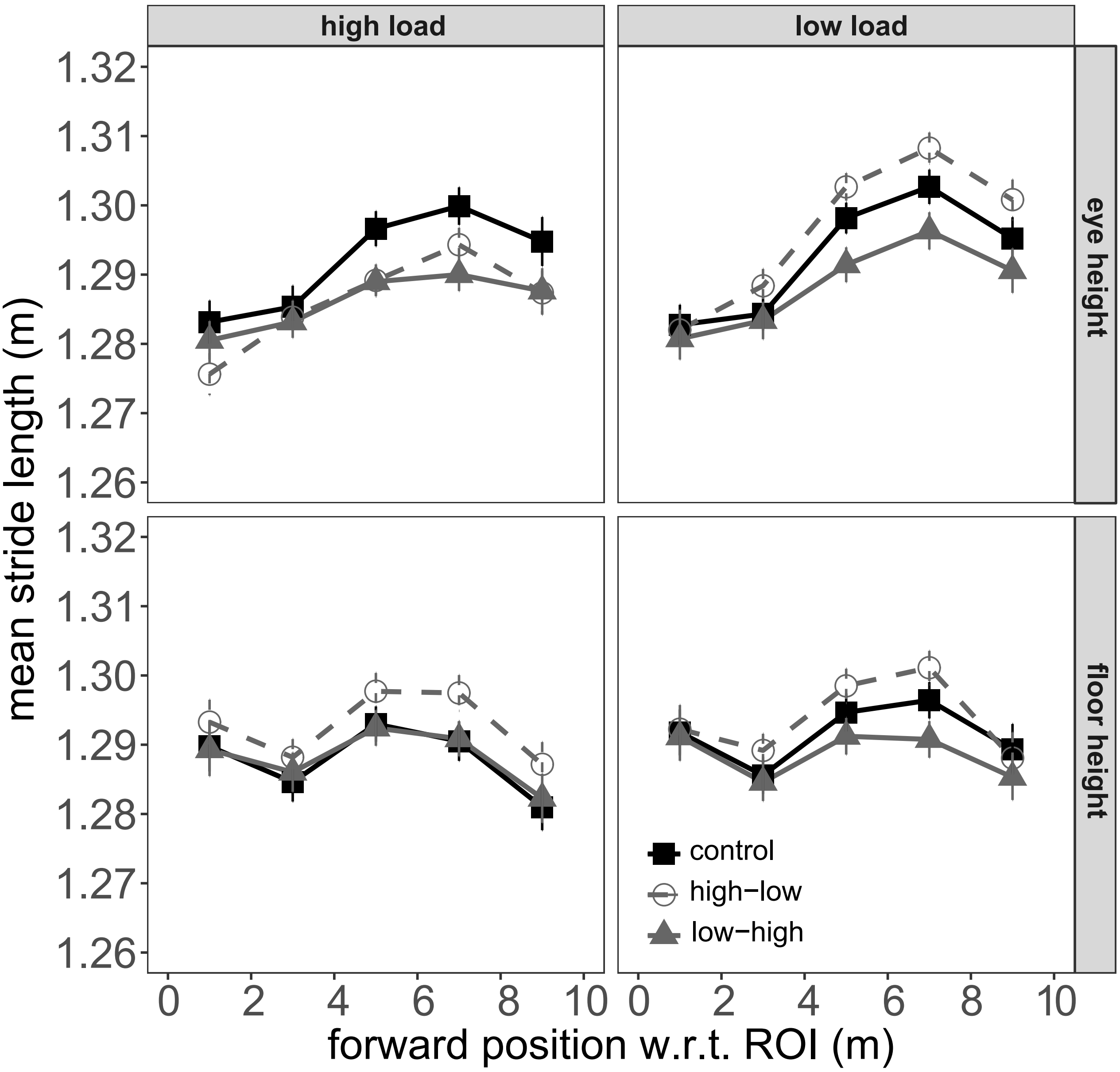

Figure S2: Average mean stride length as a function of position on the walkway, separately for different combinations of floor patttern, perceptual load and discrimination target height. Error bars are within-subject standard errors of the mean.

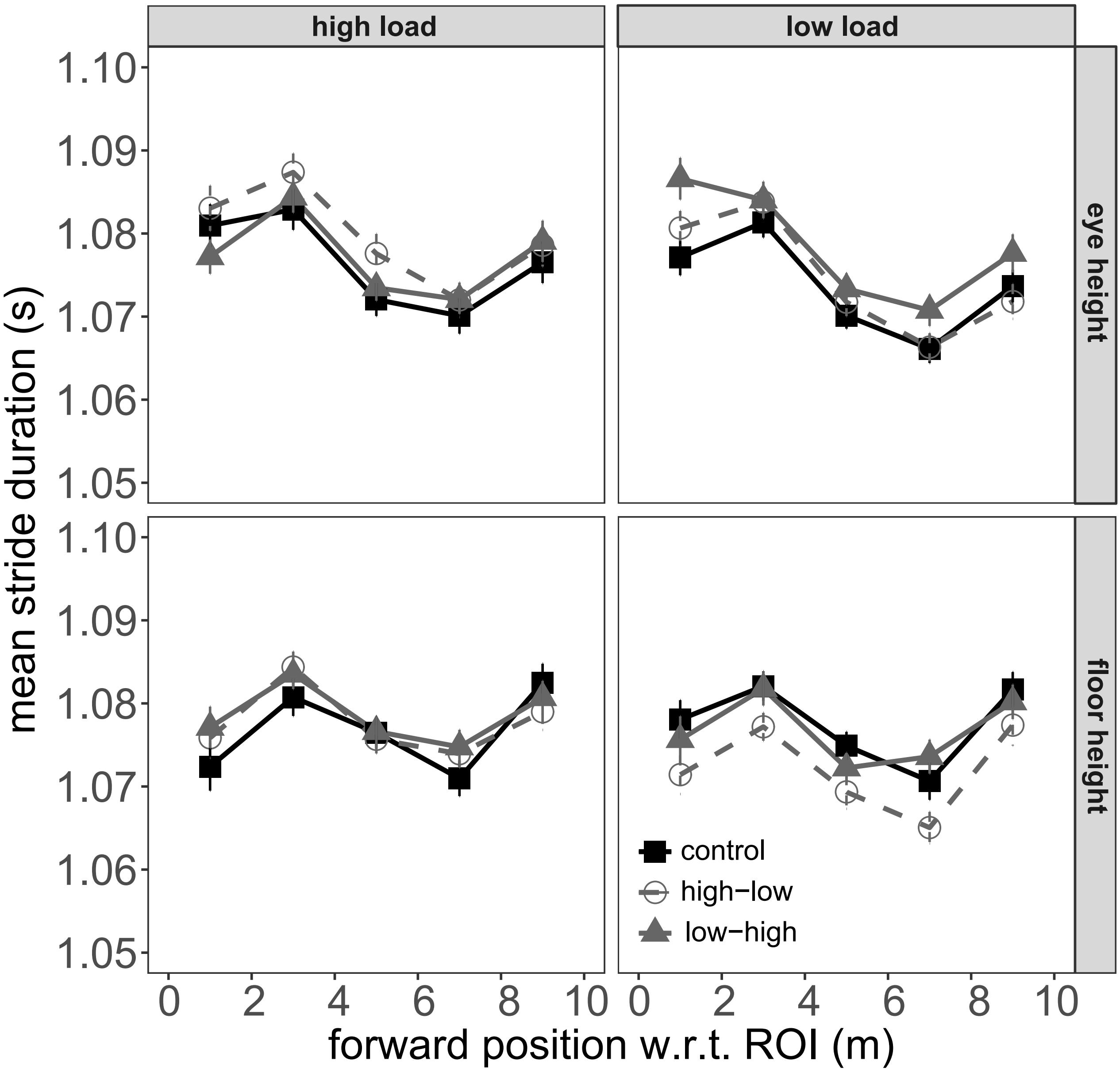

Figure S3: Average mean stride duration. Conventions as in Figure S2.
